# Surgical treatment of left ventricular aneurysm in patients with prior myocardial infarction in the absence of obstructive coronary artery disease (MINOCA): a cohort study

**DOI:** 10.1186/s13019-023-02385-0

**Published:** 2023-10-31

**Authors:** Yangwu Song, Fei Xu, Wei Feng

**Affiliations:** Department of Cardiovascular Surgery, National Clinical Research Center of Cardiovascular Diseases, Fuwai Hospital, National Center for Cardiovascular Diseases, Chinese Academy of Medical Sciences and Peking Union Medical College, Beijing, China

**Keywords:** Surgical treatment, Left ventricular aneurysm, Myocardial infarction, Aneurysmectomy, MINOCA

## Abstract

**Background:**

There is a paucity of studies examining the treatment of patients with prior myocardial infarction in the absence of obstructive coronary arteries (MINOCA) and with a concomitant left ventricular aneurysm. Our study aims to illustrate the clinical characteristics and report the mid-term surgical outcomes in this distinct entity.

**Methods:**

Ten patients with MINOCA and left ventricular aneurysm were investigated. The MINOCA was diagnosed according to Scientific Statement from the American Heart Association. The indication for left ventricular reconstruction was as follows: clear evidence of both an aneurysmal and akinetic left ventricle with a history of myocardial infarction accompanied by heart failure symptoms, angina, or ventricular arrhythmias. Major adverse cardiovascular and cerebrovascular events (MACCE), including death, myocardial infarction, stroke was considered the primary endpoints.

**Results:**

The median follow-up for the whole study population was 64.5 months. Seven MINOCA patients developed a left ventricular aneurysm within 4 years and three MINOCA patients were found to have a concomitant left aneurysm at the first admission. Before surgery, no patients were prescribed angiotensin-converting enzyme inhibitors. Statins, dual antiplatelet therapy, and β-blockers were prescribed in 2, 5, and 5 patients, respectively. After surgery, no MACCE occurred in the follow-up. There was a significant increase in ejection fraction (EF) in the follow-up (p = 0.0009).

**Conclusions:**

Close monitoring and standard medical treatment are required before a left ventricular aneurysm occurs in MINOCA patients. Left ventricular reconstruction remains a viable option for MINOCA patients with left ventricular aneurysms and mid-term outcomes were satisfying in this distinct entity.

## Background

It is increasingly identified that a group of myocardial infarction patients presents with no angiographic obstructive coronary artery disease (≥ 50% diameter stenosis in a major epicardial vessel) and the term myocardial infarction with non-obstructive coronary arteries (MINOCA) has been coined for this distinct entity [1, 2]. The European Society of Cardiology [1] developed the first international position article on MINOCA and proposed the following MINOCA criteria: (1) acute myocardial infarction criteria as defined by the “Third Universal Definition of Myocardial Infarction” [3]; (2) non-obstructive arteries with no lesions ≥ 50% in a major epicardial vessel; and (3) no other clinically overt specific cause that can serve an alternative cause for the acute presentation. Due to the limitation of the troponin bioassay, the “Fourth Universal Definition of Myocardial Infarction” recently differentiated myocardial infarction from myocardial injury [4]. With this revised concept of acute myocardial infarction, the American Heart Association stated that the term MINOCA should refer to patients in whom there is an ischemia basis for their clinical presentation [5].

Issues remain that, unlike acute myocardial infarction, there is a paucity of dedicated studies examining MINOCA and therefore a lack of evidence-based therapies in these patients. In the scientific statement from the American Heart Association, selective and empirical therapy is proposed in the management strategy for patients with MINOCA [5]. However, there is a small proportion of patients in whom left ventricular aneurysm occurs, refractory to medical treatment and even implantable cardioverter defibrillator. And only small sample size cohort literature and case report exist to address this conundrum [6–9].

Our study aims to (1) illustrate the clinical characteristics in patients with prior myocardial infarction and left ventricular aneurysm in the absence of obstructive coronary artery disease, and (2) report the mid-term surgical outcomes in this distinct entity.

## Methods

### Study design

The data were retrospectively collected in the Information Center of Fuwai Hospital. The Registry contains clinical data of all the patients referred for left ventricular reconstruction from 1999 to 2017. The institutional review board at Fuwai Hospital approved the use of clinical data, and waived individual informed consent for this study.

### Patient population

**Fig. 1:**
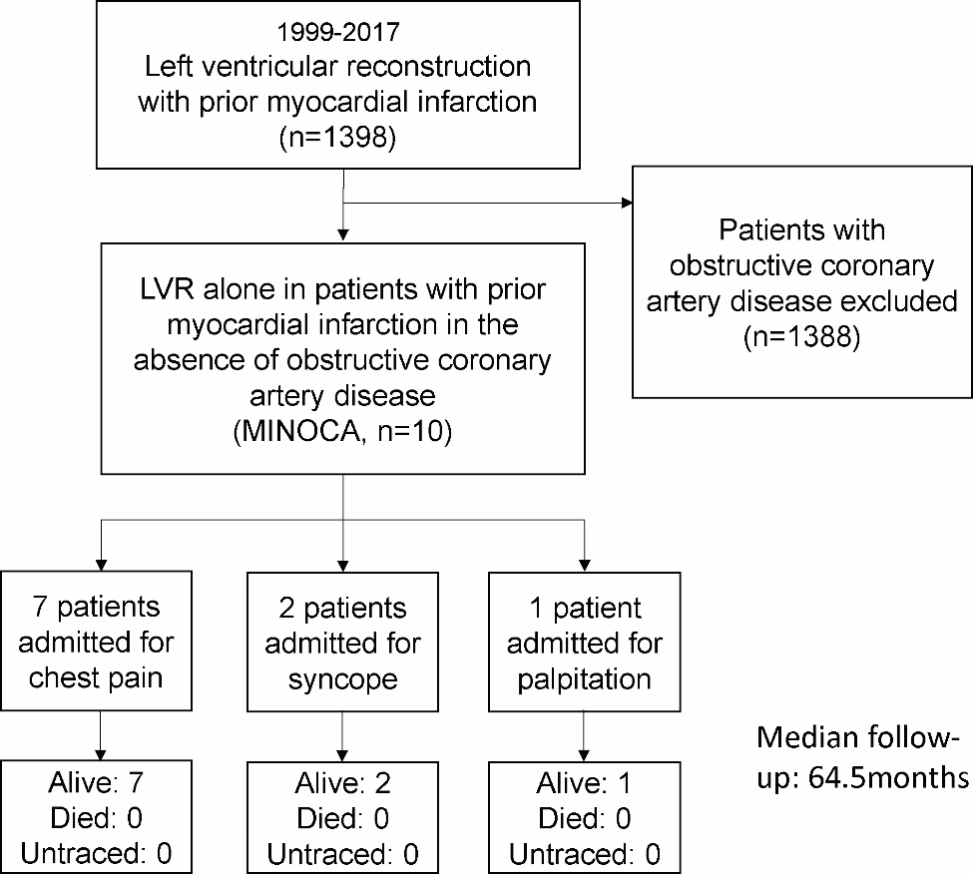
Flow chart of the study

Figure 1 shows the flow chart of our study. Among 1398 patients undergoing left ventricular reconstruction (LVR), there were 10 patients with prior myocardial infarction in the absence of obstructive coronary artery on preoperative angiogram. These 10 patients constituted the target population in this study. The operative indication for LVR was the presence of both of the following criteria: (1) there was clear evidence of aneurysmal and akinetic left ventricle according to echocardiography, left ventriculography, or magnetic resonance imaging. (2) patients presented symptoms of heart failure, refractory angina, thromboembolism, a concomitant structural cardiac disease requiring surgery or ventricular arrhythmias which were refractory to medical treatment, implantable cardioverter defibrillator, and endocardial ablation [10].

### Diagnosis

The MINOCA was diagnosed according to the 2019 Scientific Statement from American Heart Association as follows [5]. [1] Acute myocardial infarction (modified from the “Fourth Universal Definition of Myocardial Infarction” Criteria); [2] Nonobstructive coronary arteries on angiography. [3] No specific alternate diagnosis for the clinical presentation.

In our patient population, all patients (n = 10) had a prior myocardial infarction and this was confirmed in the pathological examination of the fibrous scar tissue removed during left ventricular reconstruction. Preoperative coronary angiography revealed nonobstructive coronary arteries. Alternative diagnosis, such as Takotsubo Syndrome, cardiomyopathy and myocarditis has been excluded according to magnetic resonance imaging. Thus, a working diagnosis of MINOCA can be made.

### Data collection and definitions

Baseline information was collected and definitions of pre-operative characteristics conform to those of the STS database(www.sts.org). Trained staff was in charge of the institutional database collection. Two professional typists worked independently. The echocardiographic assessment was made by transthoracic echocardiography preoperatively and before discharge. The severity of mitral regurgitation (MR) was graded on the basis of color Doppler images as follows: 1 = mild, 2 = moderate, 3 = moderate-to-severe, and 4 = severe [11].

### Surgical technique

The heart was arrested, with antegrade crystalloid or cold blood cardioplegia introduced. An incision parallel to the left anterior descending artery was made in the infarcted anterior wall segment. The thrombus was carefully inspected and thoroughly removed. Two types of LVR, linear ventriculoplasty and modified left ventricular reconstruction (modified VR) were used depending on surgeon preference. The modified VR has been previously described [12]. In brief, an endoventricular purse-string suture was placed with a 1 − 0 prolene line suture. The suture was placed in the scarred tissue above the junctional zone between the normal myocardium. The suture was tied with an opening created about 2 cm. The ventricular chamber was reduced and kept in satisfactory geometry. The next closure was similar to standard linear closure. The ventriculotomy was closed with 1 − 0 prolene line as heavy horizontal mattress sutures buttressed in polytetrafluoroethylene felt and reinforced by continuous sutures. The cone shape of the cardiac apex could be restored after the suture was drawn tight and tied. The level of suture was adjusted continually to keep the ventricle from being distorted during closure and the left anterior descending artery was kept intact.

### Follow-up

According to institutional follow-up protocol, patients discharged alive were required to visit our outpatient clinic six months after surgery, and then once every year. If the patients reported adverse events, the medical records of the patients in the outpatient clinic of Fuwai Hospital were doubled checked. If the patients visited another hospital, patients were required to send their copies of medical records by mail. The median follow-up for the whole study population was 64.5 months.

### Statistical analysis

Baseline patient characteristics are represented as means with standard deviations for continuous variables and proportions for categorical variables. Changes in left ventricular ejection fraction and left ventricular end-diastolic dimension were calculated using the paired t-test.

## Results

### Baseline characteristics of patients

In the study objects, the average age of patients with MINOCA and left ventricular aneurysm was 36.46 years and 7 patients were female (Table 1). MINOCA patients had a low prevalence of cardiovascular risk factors. Three patients had ventricular arrhythmia before the first admission.

**Table 1:** Baseline characteristics

Variable	MINOCA with aneurysm (n = 10)	MI-CAD with aneurysm (n = 1388)
Age	36.46$\pm$15.74	58.37$\pm$9.45
Female	7 (70.0%)	344 (14.52%)
BMI	22.67$\pm$5.08	25.26$\pm$3.20
Smoking	4 (40.0%)	791 (64.15%)
Hypertension	3 (30.0%)	628 (50.93%)
Hypercholesterolemia	3 (30.0%)	668 (54.18%)
Diabetes mellitus	0 (0.0%)	297 (24.09%)
Prior stroke	0 (0.0%)	172 (13.95%)
Prior atrial fibrillation	0 (0.0%)	41 (3.33%)
Preoperative serum creatinine (umol/L)	72.48$\pm$21.52	91.99$\pm$28.55
NYHA class		
I	1 (10%)	37 (3.00%)
II	5 (50%)	678 (54.99%)
III	2 (20%)	443 (35.93%)
IV	2 (20%)	75 (6.08%)
Preoperative LVEDD (mm)	53.70$\pm$6.27	58.55$\pm$7.19
Preoperative LVEF	52.38$\pm$6.31	44.00$\pm$9.41
Preoperative MR grade		
0	6 (60%)	520 (43.17%)
1	3 (30%)	210 (17.03%)
2	1 (10%)	457 (36.58%)
3	0	31 (2.51%)
4	0	15 (1.22%)

*BMI*, body mass index; *LVEDD*, left ventricular end-diastolic dimension; *LVEF*, left ventricular ejection fraction; *MI-CAD*, myocardial infarction with coronary artery disease; *MINOCA*, myocardial infarction with non-obstructive coronary arteries; *MR*, mitral regurgitation; *NYHA*, New York Heart Association

### Presenting symptoms

Table 2 shows the clinical course for surgically treated patients with MINOCA and indications for each patient. Seven MINOCA patients (Patient No. 1–7) developed a left ventricular aneurysm within 4 years and three MINOCA patients (Patient No. 8–10) were found to have a concomitant left aneurysm at the first admission. Patients were divided into three groups according to their onset of symptoms, including chest pain (No. 1, 2, 3, 4, 5, 6, 7), syncope (No. 8, 9), and palpitation (No. 10).

**Table 2:** Clinical course for surgically treated patients with MINOCA

No. of Patient	Age at Diagnosis of MINOCA with no aneurysm	Medical treatment after the diagnosis of MINOCA	Age at Diagnosis of MINOCA with LV aneurysm	Size of left ventricular aneurysm (mm)	Indication for surgery	Type of LVR	Duration of follow-up (months)
1, F	71	Aspirin, nitrates, β-blockers, statins, digoxin and diuretics	71	45*30	Ventricular septal defect, refractory angina and heart failure	LR	22
2, M	32	Aspirin, β-blockers	35	50*29	Refractory angina	LR	76
3, F	23	Traditional Chinese medicine	27	47*26	Refractory angina	Modified VR	65
4, F	26	Aspirin	26	34*24	Refractory angina, left ventricular thrombus	Modified VR	87
5, F	30	Aspirin, β-blockers and statins	33	49*21	Heart failure	Modified VR	100
6, M	36	None	40	40*22	Ventricular arrhythmia	Modified VR	64
7, M	45	Nitrates, β-blockers	46	44*20	Refractory angina	Modified VR	101
8, F		None.	17	45*21	Ventricular arrhythmia	Modified VR	49
9, F		None	45	40*30	Ventricular arrhythmia and thrombus formation	LR	33
10, F		None	24	30*30	Ventricular arrhythmia, moderate aortic valve regurgitation, subaortic membrane	Modified VR	22

*F*, female; *LR*, linear repair; *M*, male; *MINOCA*, myocardial infarction in the absence of obstructive coronary artery disease; *Modified VR*, modified ventricular reconstruction

**Fig. 2:**
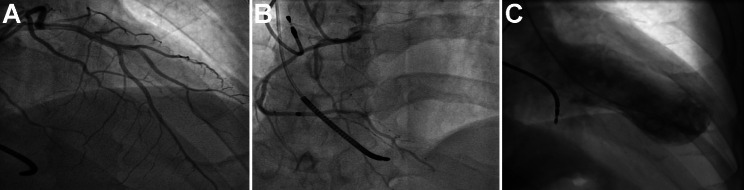
Coronary angiography and left ventriculography. (**A**). The normal left coronary artery. The patient received an implantable cardioverter defibrillator due to ventricular arrhythmias refractory to medical treatment. (**B**). The normal right coronary artery. (**C**). Left ventriculography shows the apical aneurysm

Figure 2 shows the coronary angiography and left ventriculography of patient No. 6. They did not have any comorbidity nor prior psychiatric stress. Readmission to hospital occurred in all seven patients after several months due to heart failure (No. 1, 6), refractory angina (No. 1, 2, 4, 8), and ventricular arrhythmia (No. 7). Left ventricular reconstruction was performed accordingly.

**Fig. 3:**
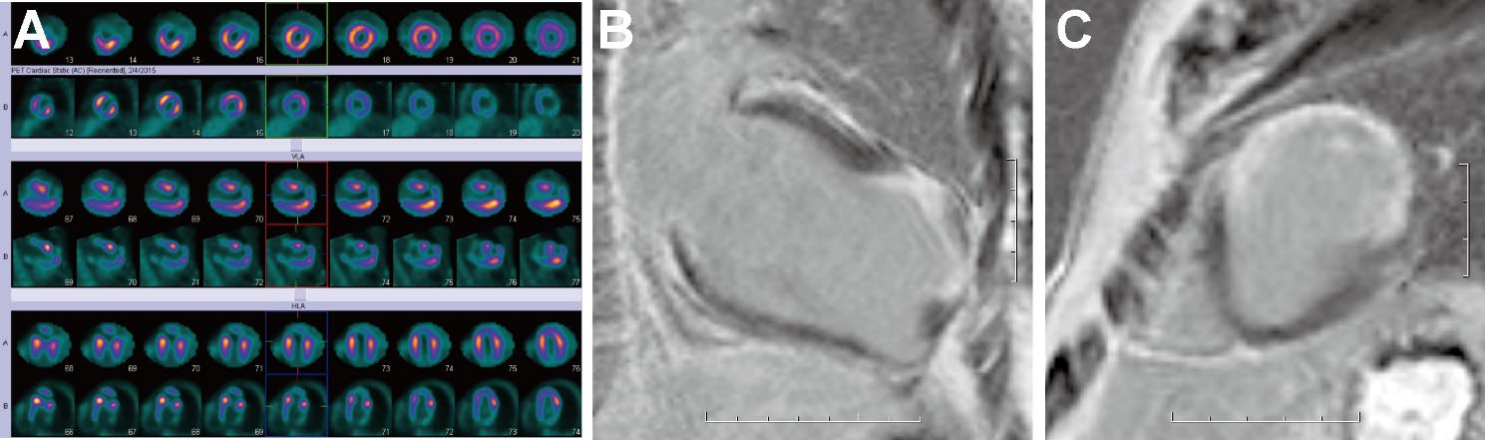
Single-Photon Emission Computed Tomography (SPECT), 18 F-fluorodeoxyglucose (18 F-FDG) myocardial metabolic imaging, and magnetic resonance imaging (MRI). (**A**). SPECT and 18 F-FDG myocardial metabolic imaging demonstrated impaired blood perfusion and metabolism in the apex and part of apical segments of anterior wall of the left ventricle. (**B**), (**C**). The myocardial delayed-enhanced scan showed transmural late gadolinium enhancement (LGE) of the thinned area above

**Fig. 4:**
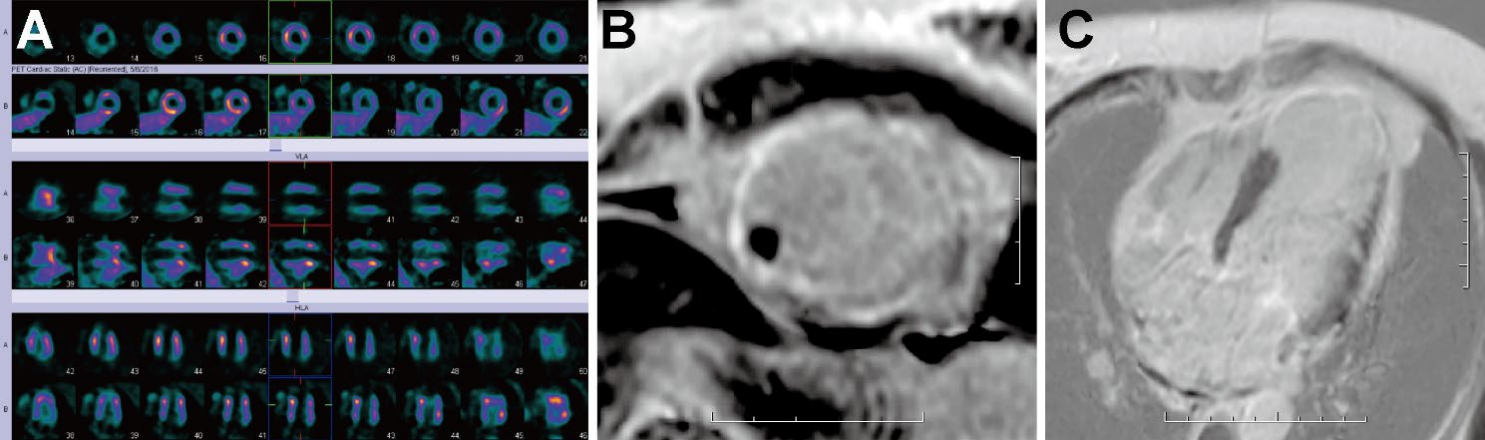
(**A**). Rest ^99m^Tc-MIBI myocardial SPECT images showed myocardial perfusion defects in the apex and inferior basal segment of the left ventricle. 18 F-FDG myocardial metabolism images revealed metabolic defects. (**B**), (**C**). The ventricular aneurysm accounted for about one-quarter of the left ventricular cavity. First-pass perfusion imaging demonstrated perfusion defect in the above segments. An intracardiac mural thrombus was observed

Neurogenic syncope was excluded in Patient No. 8 and 9. Patient No. 8 presented with ventricular tachycardia. Pathological examination revealed that scar tissue corresponded to transmural myocardial infarction. Patient No. 9 had left ventricular thrombosis concomitantly and presented with ventricular tachycardia. Figures 3 and 4 illustrates the imaging findings of Patient No. 8 and Patient No. 9 respectively.

The electrocardiogram of Patient No. 10 result showed ventricular arrhythmia. She was prescribed antiarrhythmic drugs (amiodarone) to which she was irresponsive. The patient was transferred to our hospital for further therapy. The echocardiographic showed a left ventricular aneurysm, moderate aortic regurgitation, and subaortic membrane. Coronary angiography failed to show any significant stenosis in the major epicardial vessels. After consultation with a cardiologist and cardiac surgeon, the patient was transferred to the Adult Cardiac Surgery Institute for left ventricular aneurysmectomy, subaortic membrane excision, and aortic valve repair. Pathological examination revealed that scar tissue corresponded to transmural myocardial infarction.

### Mitral regurgitation

Patient No. 4, 5, 6 presented with mild mitral regurgitation. Patient No. 1 presented with moderate mitral regurgitation. The mitral regurgitation was left untreated.

### Echocardiographic outcomes

**Fig. 5:**
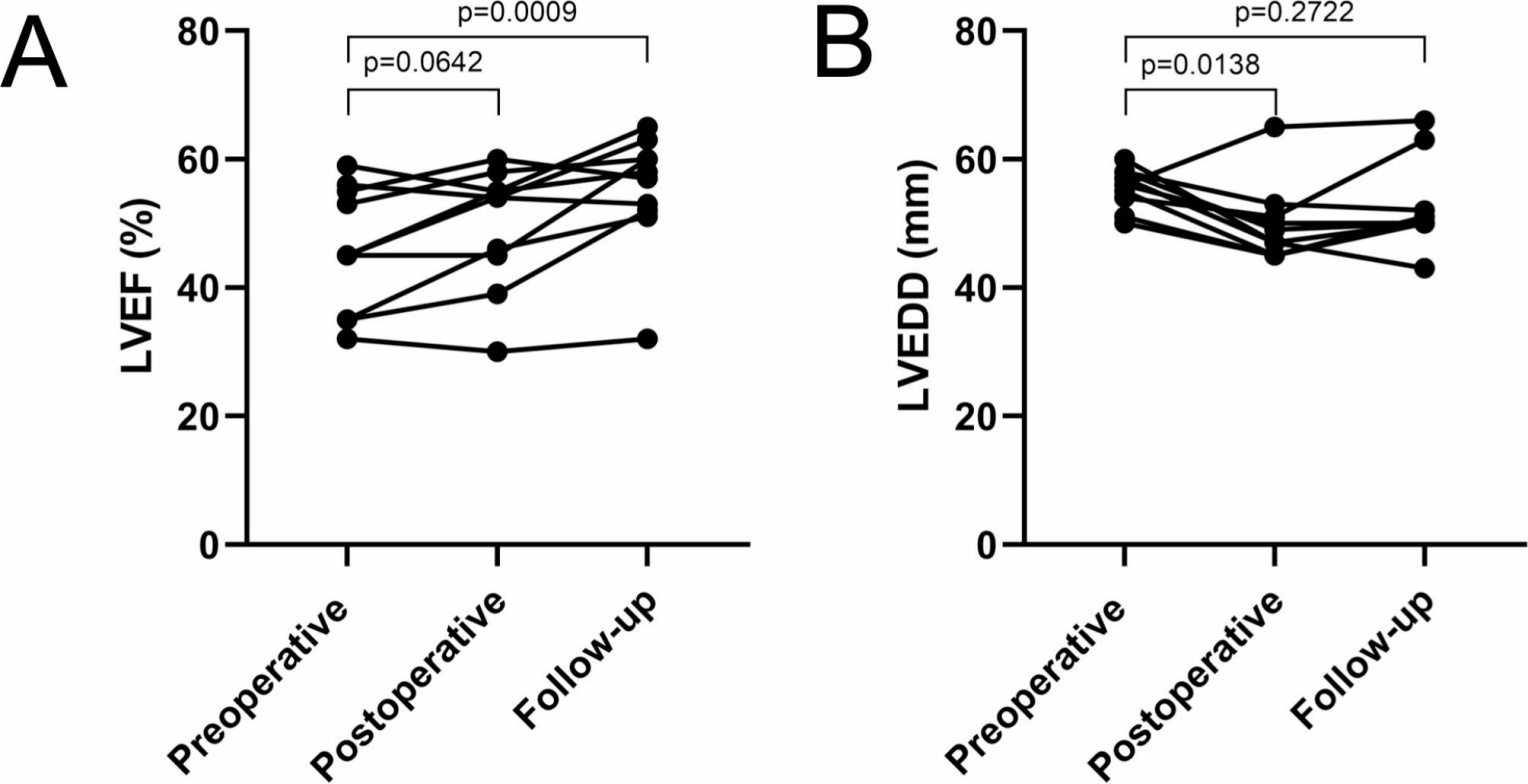
Echocardiographic outcomes preoperatively, postoperatively, and in the follow-up. EF, ejection fraction; LVEDD, left ventricular end-diastolic dimension

The mean echo follow-up time in these patients were 23 months. Figure 5 illustrated echocardiographic outcomes preoperatively, postoperatively, and in the follow-up. The aneurysms were all located in the apex of the left ventricle. No significant difference was obtained between follow-up left ventricular diastolic dimension (LVEDD) and preoperative LVEDD (p = 0.2722).

### Mid-term clinical outcomes

No major adverse cardiovascular and cerebrovascular events occurred in the follow-up including death, stroke, myocardial infarction. There was no reoccurrence of symptoms in the follow-up. However, one patient (No. 2) reported supraventricular arrhythmia three years after left ventricular reconstruction, and radiofrequency ablation was performed for this patient.

## Discussion

To the best of our knowledge, this is the most substantial series assembled to date to clarify the presentation, clinical profile, and prognosis in this underrecognized and incompletely described subgroup of patients with MINOCA developing a left ventricular aneurysm. Our main findings were that patients with MINOCA (i) developed left ventricular aneurysm within a relatively long duration; (ii) could seek surgical treatment if left ventricular aneurysm occurred; (iii) achieved satisfying outcomes after surgery with no reoccurrence of symptom and no adverse event in the follow-up.

### Characteristics of MINOCA and the key role of cardiac magnetic resonance

The demographic and clinical characteristics of MINOCA patients differ from other patients with acute myocardial infarction. In our study, compared to another 1388 patients with left ventricular aneurysm and obstructive coronary artery disease of our registry, most patients (70%) were female and the prevalence of traditional coronary artery disease risk factors were less frequent in MINOCA patients. This is in accordance with previous studies [13, 14], although this has not been consistently observed in all studies [15].

The most common causes of MINOCA are represented by plaque disruption, coronary artery spasm, coronary microvascular dysfunction, coronary embolism/thrombus, spontaneous coronary artery dissection, supply-demand mismatch [5]. Multiple diagnostic pathways have been proposed to evaluate these underlying causes [16]. Among them, cardiac magnetic imaging has been regarded as a central role because of its ability to exclude the clinically overt causes and clinically subtle non-ischemic mechanisms of myocyte injury and to provide a diagnosis of myocardial infarction [5, 17]. It was reported that cardiac magnetic resonance identifies the underlying cause in as many as 87% of patients with MINOCA [18]. It is the method of choice to distinguish myocardial infarction from myocarditis, cardiomyopathy, and takotsubo syndrome [19, 20]. In our study, one patient (Patient No. 1) had mild luminal irregularities (angiographic stenosis < 30% stenoses). The other nine patients had normal arteries, and thus plaque disruption may not be the underlying cause. Three patients (Patient No. 8, 9, 10) were found to have left ventricular aneurysms when they were first admitted to the hospital. Cardiac magnetic resonance and SPECT were performed to provide a diagnosis of myocardial infarction and to exclude non-ischemic mechanisms of myocyte injury such as Takotsubo syndrome, cardiomyopathies, and myocarditis.

### Management strategies of MINOCA

There is a well-established body of literature regarding the management of acute myocardial infarction with obstructive coronary artery disease [21, 22]. However, there is a paucity of dedicated studies examining management strategies of MINOCA and no published randomized controlled trial data are available to inform clinicians on best practices [5]. The article by Lindahl provides the first insight into potential long-term medical therapy in the management of MINOCA [2]. In this study, it was revealed that patients treated with statins and renin-angiotensin system blockers had a significantly 23% and 18% lower risk of a major adverse cardiac event during follow-up. In contrast, there were no significant reductions in the risk of major adverse cardiac events after treatment with β-blockers and dual antiplatelet therapy. The MINOCA BAT (Randomized Evaluation of β-blocker and Angiotensin-Converting Enzyme Inhibitor/Angiotensin Receptor Blocker Treatment in MINOCA Patients) study was started in 2018, and estimated to complete in 2026 [23]. In our study, great variability exists in the manner in which patients with MINOCA were evaluated and treated. Renin-angiotensin system blocker was administered in no patient and 2 patients (20%) were prescribed statins. A lower prescription rate of secondary prevention regimen could predispose patients with MINOCA to adverse left ventricular remodeling after myocardial infarction and therefore could account for left aneurysm formation in such a relatively longer duration, although this hypothesis has not been verified.

### Management of MINOCA accompanied by left ventricle aneurysm

Evidenced-based treatments for MINOCA are lacking since there is no published randomized clinical trial on MINOCA, although there is one ongoing randomized clinical trial [24]. A small proportion of patients with MINOCA developed left ventricular aneurysm. Only case reports exist to address this conundrum. The therapeutic strategies vary from optimal medical treatment [9] to left ventricular reconstruction [7, 8] to epicardial ablation [6]. The indication for surgery in our patient series was consistent with Li B’s [7] and Takaseya’s reports [8]. However, our small sample size does not suffice for statitical analysis. Thus, in this subgroup of MINOCA patients, a body of evidence is urgently warranted in the future.

LVR is an operation applied to patients with congestive heart failure and ventricular dilation following infarction [25]. This surgical procedure reduces ventricular size by excluding the scarred segment and the increase in left ventricular ejection fraction would be expected [26]. LVR also rebuilds a more normal elliptical architecture [27]. Effectiveness of LVR was demonstrated that in > 5000 patients in observational studies [28]. However, the surgical treatment for ischemic heart failure (STICH) trial concluded that the addition of LVR to coronary bypass grafting did not lead to improved survival in patients with dilated ischemic cardiomyopathy. STICH reported no history of infarction in 13% of patients and only 58% had akinesia or dyskinesia in the report by Zembala [29]. Furthermore, LVR in STICH reduced left ventricular volume only 19%, significantly lower than what is reported in multiple observational data registries [28]. This inclusion is different from the original grant submission (volume reduction > 30%). These may give rise to the discrepancies between the STICH and observational studies. In our study, a large left ventricular aneurysm in a symptomatic patient with prior myocardial infraction, particularly on the angina pectoris but also in one with heart failure, is an indication for operation. Linear ventriculoplasty and modified left ventricular reconstruction (modified VR) were used depending on surgeon preference.

Malignant ventricular tachycardia occurs most frequently in patients with coronary artery disease who have had a previous myocardial infarction and in whom a ventricular aneurysm subsequently develops in the scarred section of myocardium [30]. Approaches to control refractory ventricular tachycardia surgically in patients with left ventricular aneurysm followed by the disappearance of ventricular tachycardia in resected left ventricular aneurysm was first observed by couch [31]. The most favorable results in the surgical treatment of arrhythmogenic ventricular aneurysms have been achieved by mapping-guided subendocardial resection in combination with aneurysmectomy [32, 33]. In Rajasinghe’s study, six patients with sustained ventricular tachycardia and left ventricular aneurysm were treated surgically. Either the large ventriculotomy incision was closed primarily or a Dacron patch was used to reconstruct the left ventricle. 5 of 6 patients had postoperative electrophysiologic studies demonstrating no inducible ventricular tachycardia and remain without antiarrhythmic therapy in follow-up extending from 29 months to 86 months [30]. Similar results were also observed in Aizawa’s study [34]. Our study was consistent with the studies above. There was no reoccurrence of symptoms in the follow-up. This suggested that surgical therapy may be undertaken with low recurrence of ventricular arrhythmias during the follow-up. Future studies are warranted to elucidate the efficacy of surgical therapy in the treatment of ventricular arrhythmias.

### Prognosis of patients presenting with MINOCA and left ventricular aneurysm

Most studies have shown better outcomes than their acute myocardial infarction-obstructive coronary artery disease counterparts [13, 35, 36]. However, there were only case reports reporting the prognosis of patients with MINOCA and left ventricular aneurysm, with different management strategies implemented [6–9]. In our series, surgical indications for patients without obstructive coronary arteries were accordant with those with obstructive coronary arteries. Similar to their studies, satisfying outcomes were obtained with no major adverse cardiovascular and cerebrovascular events occurring. Additionally, no patient reported the reoccurrence of symptoms, and the aneurysm was located at the apex of the left ventricle. This suggested that the target lesion responsible for MINOCA could be limited to a single-vessel coronary artery and was either removed by the surgery or silent during the follow-up.

### Limitation

First, this was a retrospective observational study. Therefore, there is the potential for selection bias. Second, additional investigation including intravascular ultrasound and optical coherence tomography was not protocol-driven. Thus, it was unlikely to confirm the underlying cause of MINOCA. However, cardiac magnetic resonance was performed for every patient. And this could ensure that our patient cohort was pure MINOCA excluding myocarditis, cardiomyopathy, and Takotsubo syndrome. Third, medical therapy was not standardized according to current evidence-based therapies. However, there was no randomized data available to date.

## Conclusion

MINOCA can occur with multiple symptoms and lead to a left ventricular aneurysm. Thus, MINOCA patients require close monitoring and standard medical treatment. Left ventricular reconstruction remains a viable option for MINOCA patients with left aneurysms and mid-term outcomes were satisfying in this distinct entity.

## Data Availability

The data used to support the findings of this study are available from the corresponding author upon request.

## Abbreviations

CABG: Coronary artery bypass grafting
LVEDD: Left ventricular end-diastolic dimension
LVEF: Left ventricular ejection fraction
LVR: Left ventricular reconstruction
MINOCA: Myocardial infarction in the absence of obstructive coronary artery disease
